# Variants from *GIPR*, *TCF7L2*, *DGKB*, *MADD*, *CRY2*, *GLIS3*, *PROX1*, *SLC30A8* and *IGF1* Are Associated with Glucose Metabolism in the Chinese

**DOI:** 10.1371/journal.pone.0015542

**Published:** 2010-11-17

**Authors:** Cheng Hu, Rong Zhang, Congrong Wang, Jie Wang, Xiaojing Ma, Xuhong Hou, Jingyi Lu, Weihui Yu, Feng Jiang, Yuqian Bao, Kunsan Xiang, Weiping Jia

**Affiliations:** 1 Department of Endocrinology and Metabolism, Shanghai Jiao Tong University Affiliated Sixth People's Hospital, Shanghai, People's Republic of China; 2 Shanghai Diabetes Institute, Shanghai, People's Republic of China; 3 Shanghai Clinical Center for Diabetes, Shanghai, People's Republic of China; Aarhus University, Denmark

## Abstract

**Background:**

Recent meta-analysis of genome-wide association studies in European descent samples identified novel loci influencing glucose and insulin related traits. In the current study, we aimed to evaluate the association between these loci and traits related to glucose metabolism in the Chinese.

**Methods/Principal Findings:**

We genotyped seventeen single nucleotide polymorphisms (SNPs) from fifteen loci including *GIPR*, *ADCY5*, *TCF7L2*, *VPS13C*, *DGKB*, *MADD*, *ADRA2A*, *FADS1*, *CRY2*, *SLC2A2*, *GLIS3*, *PROX1*, *C2CD4B*, *SLC30A8* and *IGF1* in 6,822 Shanghai Chinese Hans comprising 3,410 type 2 diabetic patients and 3,412 normal glucose regulation subjects. *MADD* rs7944584 showed strong association to type 2 diabetes (*p* = 3.5×10^−6^, empirical *p* = 0.0002) which was not observed in the European descent populations. SNPs from *GIPR*, *TCF7L2*, *CRY2*, *GLIS3* and *SLC30A8* were also associated with type 2 diabetes (*p* = 0.0487∼2.0×10^−8^). Further adjusting age, gender and BMI as confounders found *PROX1* rs340874 was associated with type 2 diabetes (*p* = 0.0391). SNPs from *DGKB*, *MADD* and *SLC30A8* were associated with fasting glucose while *PROX1* rs340874 was significantly associated with OGTT 2-h glucose (*p* = 0.0392∼0.0014, adjusted for age, gender and BMI), the glucose-raising allele also showed association to lower insulin secretion. *IGF1* rs35767 showed significant association to both fasting and 2-h insulin levels as well as insulin secretion and sensitivity indices (*p* = 0.0160∼0.0035, adjusted for age, gender and BMI).

**Conclusions/Significance:**

Our results indicated that SNPs from *GIPR*, *TCF7L2*, *DGKB*, *MADD*, *CRY2*, *GLIS3*, *PROX1*, *SLC30A8* and *IGF1* were associated with traits related to glucose metabolism in the Chinese population.

## Introduction

Diabetes is one of the major health problems worldwide. According to the results of China National Diabetes and Metabolic Disorders Study, the prevalences of total diabetes and prediabetes in China were 9.7% and 15.5%, respectively [1]. Over 90% of the Chinese diabetes patients are type 2 diabetes. Type 2 diabetes is a metabolic disorder characterized by chronic hyperglycemia in the context of insulin resistance and relative insulin deficiency [2]. Although Western lifestyle contributes a lot to the type 2 diabetes epidemic, genetic determinants also influence type 2 diabetes susceptibility. Nowadays, multiple genes were identified to influence type 2 diabetes susceptibility, fasting and postprandial glucose levels [3], [4], [5], [6]. Recent reports on meta-analysis of genome-wide association studies focusing on glucose and insulin related traits identified nine novel fasting glucose loci (*ADCY5*, *MADD*, *ADRA2A*, *CRY2*, *FADS1*, *GLIS3*, *SLC2A2*, *PROX1* and *C2CD4B*), five oral glucose tolerance tests (OGTTs) 2-h glucose loci (*GIPR*, *ADCY5*, *VPS13C*, *GCKR* and *TCF7L2*) and one locus (*IGF1*) associated with fasting insulin levels and insulin resistance [7], [8]. Besides, the effects of previous reported type 2 diabetes and/or fasting glucose loci *G6PC2*, *GCK*, *GCKR*, *MTNR1B*, *DGKB*, *SLC30A8* and *TCF7L2* were also replicated in the meta-analysis [7], [8]. However, as the initial studies were performed in the European descent adults, replication studies in other ethnic samples are important to fully understand their effects on disease susceptibility. Among these loci, only the effects of *GCK*, *GCKR*, *G6PC2* and *MTNR1B* on fasting glucose levels and beta cell function had been well validated in multiple populations [9], [10], [11], [12], [13], [14], while the effects of the other loci on fasting and 2-h glucose in non-European descent populations remained largely unknown. The effects of these novel loci on type 2 diabetes risk were also unclear. In the present study, we aimed to test for the association of SNPs from fifteen reported loci and type 2 diabetes susceptibility and quantitative traits related to glucose metabolism in the case-control samples of Shanghai Chinese.

## Methods

### Ethics statement

This study was approved by the institutional review board of Shanghai Jiao Tong University Affiliated Sixth People's Hospital in accordance with the principle of the Helsinki Declaration II. Written informed consent was obtained from each participant.

### Participants

We recruited a total of 6,822 participants of Chinese Han ancestry residing in Shanghai, comprising 3,410 type 2 diabetes patients and 3,412 controls. All cases were unrelated type 2 diabetes patients recruited from the inpatient database of Shanghai Diabetes Institute. The controls were subjects with normal glucose tolerance as assessed by standard 75 g OGTTs, and with negative family history of diabetes that recruited from Shanghai Diabetes Study [15] and Shanghai Diabetes Study II [16]. The clinical characteristics of the cases and controls were shown in Table 1.

**Table 1:** Clinical characteristics of the study samples.

	Cases	Controls
Samples (*n*)	3,410	3,412
Male/female (*n*)	1,871/1,589	1,364/2,048
Age (years)	60.33±12.49	50.10±14.27
BMI (kg/m^2^)	24.38±3.51	23.46±3.25
Fasting glucose (mmol/l)	-	5.02±0.50
2-h glucose (mmol/l)	-	5.46±1.13
Fasting insulin (mmol/l)	-	37.02(26.04, 51.84)
2-h insulin (mmol/l)	-	168.03(92.16, 279.84)
HOMA-B	-	89.87(62.63, 135.84)
HOMA-IR	-	1.33(0.93, 1.90)

Data are shown as mean±SD, median (interquartile range) or *n*.

### Clinical measurements

Phenotypes for anthropometric and biochemical traits related to glucose metabolism were extensively measured for both case and control subjects. OGTTs were performed in the controls in the morning after an overnight fast. Blood samples were obtained at the fasting and 2 h during OGTTs. Plasma glucose and serum insulin were measured. Basal insulin sensitivity and beta cell function were calculated from fasting plasma glucose and insulin using HOMA [17]. In addition, insulin secretion and sensitivity were also estimated according to the indices proposed by Stumvoll et al [18] and Gutt et al [19].

### SNP selection, genotyping and quality control analysis

We selected seventeen SNPs from fifteen loci (*GIPR* rs10423928, *ADCY5* rs2877716 and rs11708067, *TCF7L2* rs12243326 and rs4506565, *VPS13C* rs17271305, *DGKB* rs2191349, *MADD* rs7944584, *ADRA2A* rs10885122, *FADS1* rs174550, *CRY2* rs11605924, *SLC2A2* rs11920090, *GLIS3* rs7034200, *PROX1* rs340874, *C2CD4B* rs11071657, *SLC30A8* rs11558471, and *IGF1* rs35767) which were recently reported to be associated with fasting or OGTT 2-h glucose levels [7], [8]. The SNPs were genotyped by using primer extension of multiplex products with detection by matrix-assisted laser desorption ionization – time of flight mass spectroscopy using a MassARRAY Compact Analyzer (Sequenom, San Diego, CA, USA). All seventeen SNPs passed genotype quality control analyses.

### Statistical analysis

The Hardy-Weinberg equilibrium test was performed before the association analysis. SNPs failed Hardy-Weinberg equilibrium tests (*p*<0.01 in the controls) were excluded. The allelic frequencies between the diabetic patients and controls were compared using *χ^2^* tests, and ORs with 95% CIs were presented. As age, gender and BMI differed in the cases and controls, we further adjusted them as confounding factors by logistic regression. Quantitative traits were analyzed by linear regression adjusted for age, gender and BMI under an additive genetic model. All skewly distributed quantitative traits, including fasting and 2-h insulin levels, HOMA-B, HOMA-IR, STUMVOLL and GUTT, were logarithmically transformed (log_10_) to approximate univariate normality (*p*>0.01 by Kolmogorov-Smirnov test). In order to adjust multiple comparison, 10,000 permutations were performed for each trait to assess empirical *p* values using PLINK [20]. The statistical analyses were performed using SAS for Windows (version 8.0; SAS Institute, Cary, NC, USA) unless specified otherwise. A two-tailed *p* value of <0.05 was considered statistically significant.

The statistic power was estimated under an additive genetic model based on the previously reported effect size and allele frequency observed in our samples. For SNPs with minor allele frequency over 0.2, our sample size had over 80% power to detect an effect size of 0.035 mmol/l for fasting glucose, 0.10 mmol/l for 2-h glucose and an OR of 1.13 for type 2 diabetes risk. For SNPs with minor allele frequency equal to 0.05, our sample size had over 80% power to detect an effect size of 0.08 mmol/l for fasting glucose, 0.17 mmol/l for 2-h glucose and an OR of 1.24 for type 2 diabetes risk.

## Results

All SNPs were in accordance with Hardy-Weinberg equilibrium. Table 2 showed the analyses of associations between these SNPs and type 2 diabetes. *GIPR* rs10423928 (OR 1.097, 95%CI 1.005–1.197, *p* = 0.0378), *TCF7L2* rs4506565 (OR 1.273, 95%CI 1.072–1.511, *p* = 0.0057), *MADD* rs7944584 (OR 1.637, 95%CI 1.327–2.020, *p* = 3.5×10^−6^), *CRY2* rs11605924 (OR 1.084, 95%CI 1.000–1.174, *p* = 0.0487), *GLIS3* rs7034200 (OR 1.103, 95%CI 1.028–1.184, *p* = 0.0062) and *SLC30A8* rs11558471 (OR 1.218, 95%CI 1.137–1.305, *p* = 2.0×10^−8^) were associated with type 2 diabetes. And *PROX1* rs340874 showed a trend towards association to type 2 diabetes in our samples (OR 1.061, 95%CI 0.989–1.137, *p* = 0.0984). However, after correction of multiple comparisons by 10,000 permutations, only the effects of *MADD* rs7944584 and *SLC30A8* rs11558471 on type 2 diabetes remained to be significant (empirical *p* = 0.0002 and 0.0001, respectively). We also tested the effects of these SNPs after adjusting for age, gender and BMI as confounding factors as they differed significantly between cases and controls. Most of the SNPs showed similar effects with or without adjustment, but the association between *CRY2* rs11605924 and type 2 diabetes was lost (OR 1.078, 95%CI 0.989–1.176, *p* = 0.0882) while *PROX1* rs340874 showed a significant association to type 2 diabetes (OR 1.082, 95%CI 1.004–1.166, *p* = 0.0391).

**Table 2:** Effects of SNPs from fifteen glucose and insulin loci on type 2 diabetes susceptibility in the Chinese population.

			Risk allele frequency							
SNP	Gene	Risk/non-risk allele	Cases	Controls	OR(95%CI)	*P* value	Empirical *P* value	OR(95%CI) #	*P* value#	Empirical *P* value #
rs10423928	*GIPR*	A*/T	0.1981	0.1838	1.097(1.005–1.197)	**0.0378**	0.4718	1.100(1.002–1.208)	**0.0462**	0.5638
rs2877716	*ADCY5*	C*/T	0.9978	0.9976	1.060(0.524–2.145)	0.8717	1	1.055(0.484–2.296)	0.8932	1
rs11708067	*ADCY5*	A*/G	0.9981	0.9973	1.376(0.673–2.810)	0.3794	0.9997	1.386(0.654–2.934)	0.3940	0.9998
rs12243326	*TCF7L2*	C*/T	0.0048	0.0027	1.778(0.990–3.192)	0.0509	0.5986	1.856(0.983–3.505)	0.0564	0.6398
rs4506565	*TCF7L2*	T*/A	0.0467	0.0370	1.273(1.072–1.511)	**0.0057**	0.0903	1.318(1.097–1.584)	**0.0032**	0.0532
rs17271305	*VPS13C*	G*/A	0.1607	0.1556	1.039(0.942–1.145)	0.4475	0.9999	1.067(0.961–1.184)	0.2278	0.9877
rs2191349	*DGKB*	T*/G	0.6643	0.6555	1.040(0.968–1.117)	0.2863	0.9957	1.048(0.970–1.131)	0.2364	0.9898
rs7944584	*MADD*	A*/T	0.9784	0.9651	1.637(1.327–2.020)	**3.5×10^−6^**	**0.0002**	1.624(1.294–2.038)	**2.9×10^−5^**	**0.0006**
rs10885122	*ADRA2A*	G*/T	0.9237	0.9197	1.057(0.931–1.198)	0.3923	0.9998	1.073(0.936–1.229)	0.3119	0.9989
rs174550	*FADS1*	T*/C	0.5919	0.5787	1.056(0.985–1.131)	0.1215	0.8887	1.039(0.965–1.119)	0.3066	0.9988
rs11605924	*CRY2*	C/A*	0.2449	0.2303	1.084(1.000–1.174)	**0.0487**	0.5821	1.078(0.989–1.176)	0.0882	0.8028
rs11920090	*SLC2A2*	A/T*	0.0114	0.0092	1.249(0.889–1.754)	0.1984	0.9741	1.407(0.975–2.031)	0.0679	0.7074
rs7034200	*GLIS3*	A*/C	0.4489	0.4247	1.103(1.028–1.184)	**0.0062**	0.0961	1.120(1.038–1.209)	**0.0037**	0.0630
rs340874	*PROX1*	C*/T	0.3918	0.3778	1.061(0.989–1.137)	0.0984	0.8166	1.082(1.004–1.166)	**0.0391**	0.5063
rs11071657	*C2CD4B*	G/A*	0.3703	0.3679	1.010(0.942–1.084)	0.7710	1	1.036(0.962–1.116)	0.3485	0.9994
rs11558471	*SLC30A8*	A*/G	0.5969	0.5487	1.218(1.137–1.305)	**2.0×10^−8^**	**0.0001**	1.263(1.172–1.361)	**1.1×10^−9^**	**0.0001**
rs35767	*IGF1*	A/G*	0.3420	0.3362	1.027(0.956–1.103)	0.4724	0.9999	1.017(0.940–1.099)	0.6813	1

*risk allele for type 2 diabetes in the European descent population.

# adjusted for age, gender and BMI.

*P* values<0.05 were shown in bold.

We then analyzed the effects of these SNPs on quantitative traits in the controls. As shown in Table 3 and Table 4, SNPs from *DGKB*, *MADD* and *SLC30A8* were significantly associated with fasting glucose (*p* = 0.0086, 0.0392 and 0.0019; empirical *p* = 0.1337, 0.4833 and 0.0329; respectively) while *PROX1* rs340874 was significantly associated with OGTT 2-h glucose (*p* = 0.0014; empirical *p* = 0.0232). The glucose-raising allele of these loci showed association to lower insulin secretion index Stumvoll (*p* = 0.0237, 0.0330, 0.0482 and 0.0333; empirical *p* = 0.3290, 0.4234, 0.5624 and 0.4272). For insulin levels, *IGF1* rs35767 showed significant association to both fasting and 2-h insulin levels (*p* = 0.0153 and 0.0073; empirical *p* = 0.2351 and 0.1105) as well as insulin secretion and sensitivity indices HOMA-B, HOMA-IR and Gutt (*p* = 0.0133, 0.0160 and 0.0035; empirical *p* = 0.1997, 0.2465 and 0.0626), while *CRY2* rs11605924 was associated with 2-h insulin levels and Gutt (*p* = 0.0117 and 0.0103; empirical *p* = 0.1727 and 0.1660). However, SNPs from *GIPR*, *ADCY5*, *TCF7L2*, *VPS13C*, *ADRA2A*, *FADS1*, *SLC2A2*, *GLIS3* and *C2CD4B* showed no association to any quantitative trait of glucose metabolism in our samples.

**Table 3:** Association between SNPs from fifteen loci and glucose and insulin levels in the Chinese normal glucose regulation subjects.

SNP	Gene	Effect allele*/other allele		Fasting glucose (n = 3,312)	2-h glucose (n = 3,312)	Fasting insulin (n = 2,309)	2-h insulin (n = 2,295)
rs10423928	*GIPR*	A/T	*Beta*	−0.0118	−0.0185	0.0074	−0.0061
			(95%CI)	(−0.0428–0.0191)	(−0.0868–0.0497)	(−0.0115–0.0263)	(−0.0318–0.0197)
			*P*	0.4536	0.5951	0.4445	0.6450
rs2877716	*ADCY5*	C/T	*Beta*	−0.0190	−0.0528	0.0657	−0.0457
			(95%CI)	(−0.2637–0.2256)	(−0.5908–0.4852)	(−0.1071–0.2385)	(−0.2814–0.1899)
			*P*	0.8788	0.8476	0.4562	0.7038
rs11708067	*ADCY5*	A/G	*Beta*	0.0736	0.0421	−0.0323	−0.0126
			(95%CI)	(−0.1452–0.2925)	(−0.4400–0.5242)	(−0.1679–0.1032)	(−0.1978–0.1725)
			*P*	0.5097	0.8642	0.6403	0.8936
rs12243326	*TCF7L2*	C/T	*Beta*	−0.0780	0.0306	0.0772	−0.0833
			(95%CI)	(−0.3081–0.1521)	(−0.4764–0.5376)	(−0.0642–0.2184)	(−0.2748–0.1082)
			*P*	0.5065	0.9058	0.2847	0.3937
rs4506565	*TCF7L2*	T/A	*Beta*	0.0209	0.0682	0.0108	0.0078
			(95%CI)	(−0.0423–0.0842)	(−0.0711–0.2074)	(−0.0275–0.0491)	(−0.0443–0.0599)
			*P*	0.5165	0.3375	0.5811	0.7684
rs17271305	*VPS13C*	G/A	*Beta*	0.0236	−0.0003	0.0048	0.0038
			(95%CI)	(−0.0098–0.0571)	(−0.0741–0.0735)	(−0.0159–0.0255)	(−0.0244–0.0319)
			*P*	0.1666	0.9936	0.6492	0.7934
rs2191349	*DGKB*	T/G	*Beta*	0.0335	0.0099	−0.0025	−0.0168
			(95%CI)	(0.0085–0.0585)	(−0.0452–0.0650)	(−0.0177–0.0128)	(−0.0375–0.0040)
			*P*	**0.0086**	0.7250	0.7524	0.1140
rs7944584	*MADD*	A/T	*Beta*	0.0673	0.0483	−0.0045	−0.0006
			(95%CI)	(0.0034–0.1312)	(−0.0926–0.1892)	(−0.0440–0.0349)	(−0.0545–0.0532)
			*P*	**0.0392**	0.5014	0.8221	0.9813
rs10885122	*ADRA2A*	G/T	*Beta*	0.0010	0.0014	0.0033	0.0070
			(95%CI)	(−0.0420–0.0440)	(−0.0936–0.0965)	(−0.0221–0.0288)	(−0.0277–0.0416)
			*P*	0.9638	0.9765	0.7963	0.6939
rs174550	*FADS1*	T/C	*Beta*	0.0149	−0.0364	−0.0095	0.0017
			(95%CI)	(−0.0093–0.0390)	(−0.0896–0.0169)	(−0.0240–0.0051)	(−0.0182–0.0216)
			*P*	0.2270	0.1808	0.2018	0.8696
rs11605924	*CRY2*	A/C	*Beta*	−0.0057	−0.0596	−0.0061	−0.0304
			(95%CI)	(−0.0338–0.0224)	(−0.1214–0.0021)	(−0.0234–0.0112)	(−0.0541−0.0068)
			*P*	0.6891	0.0586	0.4915	**0.0117**
rs11920090	*SLC2A2*	T/A	*Beta*	0.0193	−0.0239	−0.0174	0.0529
			(95%CI)	(−0.1068–0.1454)	(−0.3016–0.2537)	(−0.0944–0.0596)	(−0.0518–0.1576)
			*P*	0.7643	0.8658	0.6587	0.3220
rs7034200	*GLIS3*	A/C	*Beta*	0.0090	0.0157	−0.0099	0.0012
			(95%CI)	(−0.0153–0.0333)	(−0.0380–0.0693)	(−0.0247–0.0049)	(−0.0189–0.0213)
			*P*	0.4658	0.5675	0.1900	0.9078
rs340874	*PROX1*	C/T	*Beta*	0.0200	0.0879	0.0069	−0.0010
			(95%CI)	(−0.0045–0.0444)	(0.0341–0.1416)	(−0.0078–0.0217)	(−0.0212–0.0192)
			*P*	0.1097	**0.0014**	0.3577	0.9244
rs11071657	*C2CD4B*	A/G	*Beta*	0.0039	0.0002	−0.0029	−0.0040
			(95%CI)	(−0.0204–0.0281)	(−0.0533–0.0537)	(−0.0177–0.0119)	(−0.0242–0.0163)
			*P*	0.7550	0.9943	0.7024	0.7016
rs11558471	*SLC30A8*	A/G	*Beta*	0.0376	−0.0199	−0.0036	−0.0131
			(95%CI)	(0.0139–0.0614)	(−0.0722–0.0324)	(−0.0181–0.0108)	(−0.0328–0.0067)
			*P*	**0.0019**	0.4562	0.6215	0.1941
rs35767	*IGF1*	G/A	*Beta*	−0.0101	−0.0215	−0.0191	−0.0290
			(95%CI)	(−0.0353–0.0152)	(−0.0772–0.0342)	(−0.0346−0.0037)	(−0.0501−0.0078)
			*P*	0.4343	0.4490	**0.0153**	**0.0073**

*P* values<0.05 were shown in bold. *P* values were adjusted for age, gender and BMI.

Log transformed (log_10_) values were used for fasting and 2-h insulin levels.

*Effect allele referred to the allele for higher glucose or insulin levels reported in the European descents.

**Table 4:** Association between SNPs from fifteen loci and insulin secretion and sensitivity indices in the Chinese normal glucose regulation subjects.

SNP	Gene	Effect allele*/other allele		HOMA-B (n = 2,302)	STUMVOLL (n = 2,303)	HOMA-IR (n = 2,309)	GUTT (n = 2,291)
rs10423928	*GIPR*	A/T	*Beta*	0.0082	0.0048	0.0067	0.0033
			(95%CI)	(−0.0135–0.0299)	(−0.0046–0.0142)	(−0.0129–0.0263)	(−0.0076–0.0141)
			*P*	0.4594	0.3186	0.5035	0.5547
rs2877716	*ADCY5*	C/T	*Beta*	−0.0105	−0.0375	0.0820	−0.0033
			(95%CI)	(−0.2088–0.1877)	(−0.1236–0.0486)	(−0.0972–0.2613)	(−0.1026–0.0960)
			*P*	0.9170	0.3931	0.3699	0.9479
rs11708067	*ADCY5*	A/G	*Beta*	−0.1094	−0.0402	−0.0149	−0.0065
			(95%CI)	(−0.2648–0.0460)	(−0.1078–0.0274)	(−0.1556–0.1257)	(−0.0845–0.0716)
			*P*	0.1678	0.2439	0.8352	0.8713
rs12243326	*TCF7L2*	C/T	*Beta*	0.0273	−0.0127	0.0870	0.0146
			(95%CI)	(−0.1346–0.1892)	(−0.0829–0.0576)	(−0.0594–0.2335)	(−0.0663–0.0955)
			*P*	0.7412	0.7238	0.2442	0.7235
rs4506565	*TCF7L2*	T/A	*Beta*	−0.0113	−0.0142	0.0167	−0.0077
			(95%CI)	(−0.0553–0.0327)	(−0.0333–0.0050)	(−0.0231–0.0564)	(−0.0296–0.0142)
			*P*	0.6144	0.1482	0.4116	0.4895
rs17271305	*VPS13C*	G/A	*Beta*	0.0001	−0.0043	0.0061	−0.0053
			(95%CI)	(−0.0236–0.0238)	(−0.0145–0.0060)	(−0.0153–0.0275)	(−0.0172– 0.0066)
			*P*	0.9952	0.4160	0.5768	0.3861
rs2191349	*DGKB*	T/G	*Beta*	−0.0142	−0.0087	0.0007	0.0026
			(95%CI)	(−0.0317–0.0034)	(−0.0163−0.0012)	(−0.0151–0.0165)	(−0.0062–0.0113)
			*P*	0.1130	**0.0237**	0.9305	0.5672
rs7944584	*MADD*	A/T	*Beta*	−0.0410	−0.0214	0.0053	−0.0043
			(95%CI)	(−0.0863–0.0043)	(−0.0411−0.0017)	(−0.0356–0.0462)	(−0.0270–0.0183)
			*P*	0.0763	**0.0330**	0.7991	0.7085
rs10885122	*ADRA2A*	G/T	*Beta*	0.0149	0.0056	0.0008	0.0008
			(95%CI)	(−0.0143–0.0442)	(−0.0071–0.0182)	(−0.0255–0.0271)	(−0.0139–0.0154)
			*P*	0.3175	0.3888	0.9529	0.9185
rs174550	*FADS1*	T/C	*Beta*	−0.0125	−0.0007	−0.0084	0.0020
			(95%CI)	(−0.0292–0.0042)	(−0.0079–0.0066)	(−0.0235–0.0066)	(−0.0064–0.0104)
			*P*	0.1429	0.8537	0.2736	0.6397
rs11605924	*CRY2*	A/C	*Beta*	−0.0109	−0.0024	−0.0047	0.0130
			(95%CI)	(−0.0308–0.0091)	(−0.0111–0.0063)	(−0.0227–0.0133)	(0.0031 − 0.0230)
			*P*	0.2851	0.5867	0.6073	**0.0103**
rs11920090	*SLC2A2*	T/A	*Beta*	−0.0154	−0.0095	−0.0168	−0.0151
			(95%CI)	(−0.1037–0.0729)	(−0.0478–0.0287)	(−0.0967–0.0631)	(−0.0593–0.0291)
			*P*	0.7324	0.6253	0.6801	0.5034
rs7034200	*GLIS3*	A/C	*Beta*	−0.0072	−0.0025	−0.0098	−0.0009
			(95%CI)	(−0.0242–0.0097)	(−0.0099–0.0048)	(−0.0252–0.0056)	(−0.0094–0.0076)
			*P*	0.4030	0.5020	0.2116	0.8364
rs340874	*PROX1*	C/T	*Beta*	−0.0013	−0.0080	0.0089	−0.0045
			(95%CI)	(−0.0183–0.0158)	(−0.0154−0.0006)	(−0.0064–0.0242)	(0.0130–0.0040)
			*P*	0.8855	**0.0333**	0.2551	0.3003
rs11071657	*C2CD4B*	A/G	*Beta*	−0.0060	−0.0008	−0.0024	0.0018
			(95%CI)	(−0.0231–0.0111)	(−0.0082–0.0066)	(−0.0177–0.0130)	(−0.0067–0.0104)
			*P*	0.4920	0.8285	0.7620	0.6750
rs11558471	*SLC30A8*	A/G	*Beta*	−0.0205	−0.0073	0.0006	0.0050
			(95%CI)	(−0.0371–0.0038)	(−0.0145−0.0001)	(−0.0144–0.0156)	(−0.0034–0.0133)
			*P*	**0.0162**	**0.0482**	0.9408	0.2420
rs35767	*IGF1*	G/A	*Beta*	−0.0225	−0.0033	−0.0197	0.0133
			(95%CI)	(−0.0404−0.0047)	(−0.0110–0.0044)	(−0.0357−0.0037)	(0.0044–0.0222)
			*P*	**0.0133**	0.3998	**0.0160**	**0.0035**

*P* values<0.05 were shown in bold. *P* values were adjusted for age, gender and BMI.

Log transformed (log_10_) values were used for HOMA-B, STUMVOLL, HOMA-IR and GUTT.

*Effect allele referred to the allele for higher glucose or insulin levels reported in the European descents.

## Discussion

In the current study, we tried to replicate the effects of recently reported loci influencing quantitative traits related to glucose metabolism in a Shanghai Chinese population. To our knowledge, this is the first replication study in Asian population focusing on these loci up to now. We confirmed the association between *DGKB*, *MADD* and *SLC30A8* and fasting glucose. We also found *PROX1* was associated with 2-h glucose in our samples. The effects of *IGF1* rs35767 on fasting insulin and insulin sensitivity were also observed. However, the direction of effects was opposite to that observed in the European descent samples [7], [21]. It should also be noted that the allele frequencies of rs35767 differed between European and Chinese populations (0.15 vs 0.35 for A allele). It suggests causal variant within this locus remained to be identified.

We showed SNPs from seven loci, including *GIPR*, *TCF7L2*, *MADD*, *CRY2*, *GLIS3*, *PROX1* and *SLC30A8*, had an effect on type 2 diabetes in our samples. Most of these associations were in consistence with findings in the European descent samples, except that *MADD* rs7944584 showed an association to type 2 diabetes only in the Chinese samples. *MADD* encodes mitogen-activated protein kinase activating death domain, which interacted with tumor necrosis factor alpha receptor 1 to activate mitogen-activated protein kinase and propagate the apoptotic signal [22]. Previous study in European samples showed the glucose-raising allele of *MADD* rs7944584 was associated with elevated fasting proinsulin without altering insulin secretion [21], suggesting this locus was associated with insulin processing defects. In this study, we found the glucose-raising allele was associated with a higher risk for type 2 diabetes in the Chinese population. The underlying mechanism is not clear, but it is known the defects in insulin processing may lead to endothelium reticulum stress and finally beta cell dysfunction [23]. However, this SNP showed a negligible effect on type 2 diabetes in the European population. It is not clear whether ethnic difference played a role in the effect of this locus as poor insulin compensation ability was observed in the Asians compared with the European descent populations [24]. On the other hand, we cannot exclude the possibility that the effect of *MADD* rs7944584 on type2 diabetes was over estimated or even this association was just a positive finding by chance. Thus further replication studies in the Asian samples are needed.

In this study, we failed to replicate the associations of several variants with fasting or 2-h glucose levels. Some of these un-replicated SNPs including the ones from *ADCY5*, *TCF7L2*, *SLC2A2* and *ADRA2A* were much rarer in the Asians than they were in the European descent populations (e.g., *ADCY5*, with minor allele frequency 0.002 in the Chinese vs 0.25 in the European descents). We may not have enough statistical power to replicate the effects of some loci because of the smaller minor allele frequency. Whether there are common SNPs at these loci with effects on glucose metabolism in the Asians are unknown and remain to be investigated. There are also some SNPs lacking replication because of the smaller effect size (e.g. *C2CD4B*, with reported beta = 0.008 mmol/l per allele). However, there are still loci that failed to be replicated in our samples even though we had enough statistical power, e.g. *GIPR*, which suggests heterogeneous effects of these loci may exist in the Chinese comparing with European descent populations.

Although we analyzed these loci in relatively large samples, there are several limitations of our study. First, as multiple traits and SNPs were analyzed in the current study, we could not exclude the possibility that our findings were false positive. But considering these SNPs were originally identified in large-scale genome-wide association studies and all the traits analyzed were highly related, the impact of multiple comparisons may be limited. Second, we only analyzed the effects of these loci on insulin sensitivity and secretion in the normal glucose regulation subjects as most of the type 2 diabetes patients were receiving glucose lowering therapy. However, Heni et al showed the impact of genetic variation on insulin secretion depends on glycaemia [25], [26], what are the effects of these variants in the diabetic patients and prediabetes subjects remained unknown and to be investigated. Third, only the reported SNP(s) from each locus was analyzed in the current study. As differences exist in allele frequencies as well as linkage disequilibrium structure between Asians and European populations, detailed analyzing of more SNPs from each locus in Asian samples may help identify the causal variant.

In conclusion, we analyzed the effects of SNPs from fifteen loci recently reported to be associated with fasting and/or 2-h glucose or fasting insulin in the Chinese samples, and showed SNPs from *GIPR*, *TCF7L2*, *DGKB*, *MADD*, *CRY2*, *GLIS3*, *PROX1*, *SLC30A8* and *IGF1* were associated with traits related to glucose metabolism in the Chinese population. Moreover, our data suggest heterogeneous effects of SNPs from *MADD* and *GIPR* may exist in the Chinese population comparing with European population.
